# Data for pressure ulcers and skin infections after cochlear implantation

**DOI:** 10.1016/j.dib.2020.106295

**Published:** 2020-09-09

**Authors:** Hui-Shan Hsieh, Chee-Yee Lee, Hung-Pin Wu, Ming-Ying Zhuo, Chung-Feng Hwang

**Affiliations:** a Department of Otolaryngology, Xiamen Chang Gung Hospital, Fujian 361000, China; b Department of Otorhinolaryngology-Head and Neck Surgery, Taichung Tzu Chi Hospital, Buddhist Tzu Chi Medical Foundation, Taichung 42743, Taiwan; c School of Medicine, College of Medicine, Tzu Chi University, Hualien 97071, Taiwan; d Department of Otolaryngology, Kaohsiung Chang Gung Memorial Hospital and Chang Gung University College of Medicine, Kaohsiung 83301, Taiwan

**Keywords:** Cochlear implant, Complication, Skin flap, Pressure ulcer, irradiated

## Abstract

This article contains data concerning the research article entitled “Pressure ulcers and skin infections after cochlear implantation: A delayed yet serious issue” (Hui-Shan Hsieh, Chee-Yee Lee, Hung-Pin Wu, Ming-Ying Zhuo, and Chung-Feng Hwang) [1]. This data article reports the causes of skin flap pressure ulcer over the antenna site and protocol for the clinical managements. The patients with cochlear implant (n = 315) were enrolled. We used the National Pressure Ulcer Advisory Panel (NPUAP) pressure injury staging system to grade injury severity in all patients. The data included in this article are as follows: the clinical characteristics of patients, baselines variables between groups with and without pressure ulcer, the severity of skin flap reactions based on the NPUAP pressure injury system and corresponding interventions, related clinical details of patients with pressure ulcer, This article will be valuable for routine clinical practice as serving as a paradigm.

**Specifications Table**

**Table:** 

Subject	Otorhinolaryngology and Facial Plastic Surgery
Specific subject area	Cochlear implantation
Type of data	Table and Figure
How data were acquired	Electronic health record system
Data format	Analyzed (labeled) and Raw
Parameters for data collection	Cochlear implantation
Description of data collection	The skin flap condition was assessed and graded by a single physician with applying NPUAP pressure injury staging system [2] within regular clinical visits and detailed postoperative onset and recurrent time.
Data source location	Kaohsiung, Taiwan
Data accessibility	With the article
Related research article	Hui-Shan Hsieh, Chee-Yee Lee, Hung-Pin Wu, Ming-Ying Zhuo, and Chung-Feng Hwang, Pressure ulcers and skin infections after cochlear implantation: A delayed yet serious issue. In Press.

## Value of the Data

• The dataset included in this article will be constructive for further studies explicating complications related to skin flap in cochlear implantation.
• This dataset will benefit otologic experts, caregivers or/and parents of cochlear implant patients, and medical researchers who could utilize this data to build relative work on.
• These data might be used for further studies on reducing cochlear implant complications and better antenna design.
• Younger children especially those at preschool age are at increased risk of a skin pressure ulcer over the antenna.
• Early notification and treatment can prevent implant-threatening infection.

## 1 Data Description

**Fig. 1:**
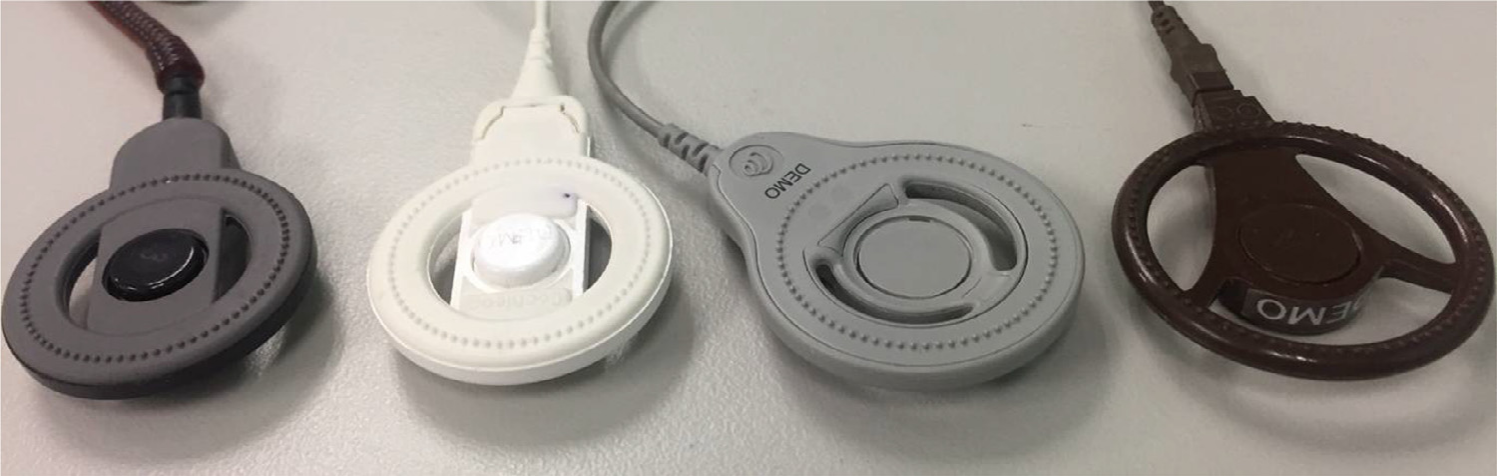
This research comprised some generations of speech processors with featured distinct antenna designs individually. From the left to right is the N6 (CP910), N5 (CP810), Freedom, and ESPrit devices, respectively.

**Fig. 2:**
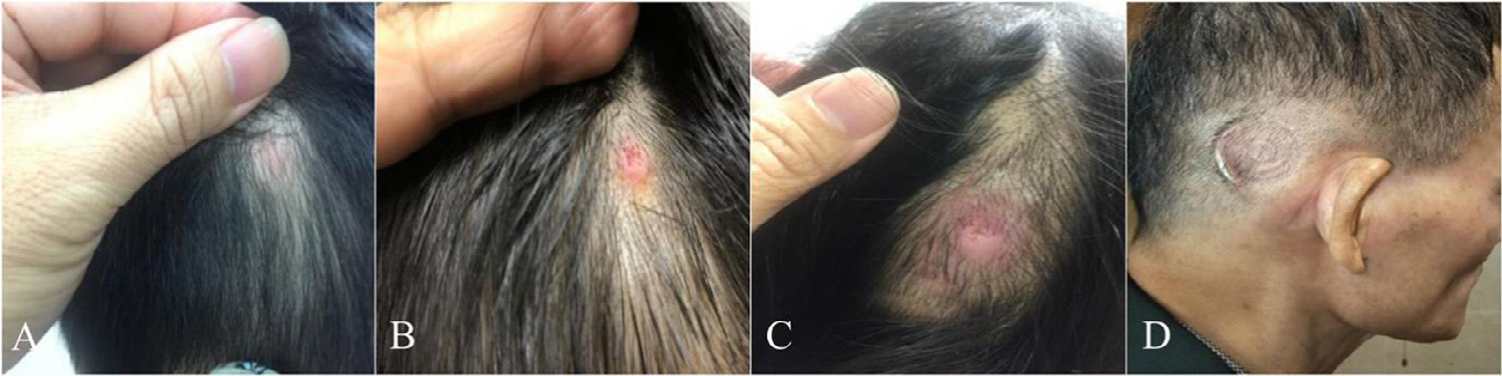
Illustration a pressure ulcer progression. (A) stage 1, (B) stage 2, (C) stage 3, and (D) stage 4.

**Fig. 3:**
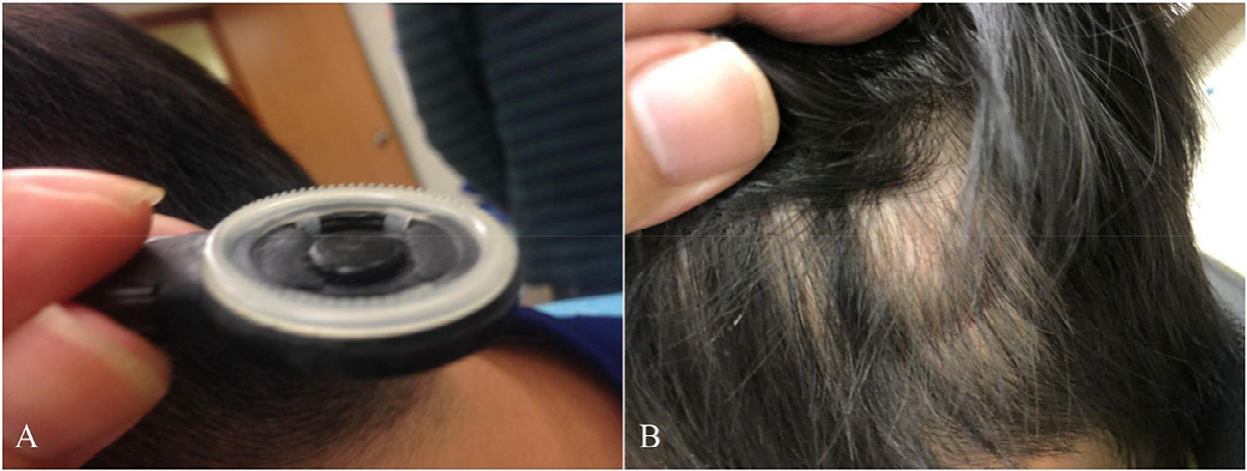
(A) A coil spacer was applied to protect a pressure injury at the center of the antenna site. (B) A pressure injury (case 15) located in the periphery of the antenna site.

This article involved data related to the research article entitled “Pressure ulcers and skin infections after cochlear implantation: a delay yet serious issue” (Hui-Shan Hsieh, Chee-Yee Lee, Hung-Pin Wu, Ming-Ying Zhuo, and Chung-Feng Hwang) [1]. Table 1 summarized the clinical characteristics of patients with cochlear implant, including surgical methods and speech processors. Following long-term clinical observations in our medical institute, we furthered identified the common characteristics of patients with skin reactions. Table 2 analysed the clinical variables between the groups with and without pressure ulcers. Moreover, we noted the causes of skin reactions were similarly to that of pressure ulcers. Table 3 detailed the severity of skin flap reactions in the antenna area according to the NPUAP staging system [2]and recommended interventions. Table 4 documented the characteristics and the postoperative onset time of patients with scalp pressure ulcers. Fig. 1 compared several generations of speech processors with distinct antenna devise, from the left to right are the N6 (CP910), N5 (CP810), Freedom, and ESPrit devices. Fig. 2 illustrated the progression of a pressure ulcer located on the external antenna site. Fig. 3 (A) utilizing a coil spacer to cover a pressure injury at the center of the antenna. (B) shows the only pressure injury event located in the periphery of the antenna (noted after 30 months of implantation). All the raw datasets in each table are enclosed in the supplementary data.

**Table 1:** Clinical characteristics of patients with cochlear implantation.

	n = 315
Sex (n, %)	
Male	172, 54.6%
Female	143, 45.4%
Age, years (mean, range)	13.17, 1-68
Without skin reaction (mean, SD)	11.82, 17.33
With skin reaction (mean, SD)	13.24, 17.05
Age, years (n, %)	
≤ 7	180, 57.1%
> 7	135, 42.9%
Methods of surgery (n, %)	
Inverted J incision	33, 10.5%
Minimal invasive incision	282, 89.5%
Speech processor (n, %)	
ESPrit	88, 27.9%
Freedom (n, %)	45, 14.3%
N5 (n, %)	105, 33.3%
N6 (n, %)	77, 24.4%

**Table 2:** Baseline variables between groups with and without pressure ulcer.

Variables	With pressure ulcer (n = 22)	Without pressure ulcer (n = 293)	P-value
Sex (n, %)			0.675
Male	11, 50%	160, 54.6%	
Female	11, 50%	133, 45.4%	
Age, years (mean ± SD)	11.82 ± 17.33	13.24 ± 17.05	0.707
Age, years (n, %)			0.048
≤ 7	17, 77.3%	163, 55.6%	
> 7	5, 22.7%	130, 44.4%	
Methods of surgery (n, %)			0.096
Inverted J incision	0, 0%	33, 11.3%	
Minimal invasive incision	22, 100%	260, 88.7%	
Speech processor (n, %)			0.011
ESPrit	0, 0%	88, 30.0 %	
Freedom	4, 18.2 %	41, 14.0 %	
N5	8, 36.4 %	97, 33.1%	
N6	10, 45.5%	67, 22.9%

**Table 3:** The severity of skin flap reactions in the area of the external antenna according to the National Pressure Ulcer Advisory Panel (NPAUP) pressure injury staging system (stages 1–4) and the recommended interventions.

Pressure ulcer stage	n = 30 (events) (n, %)	Management
Stage 1	22, 73.3%	Information on prevention (stop wearing the device for a brief period, loosen the magnet or use a coil spacer as needed). Topical antibiotics.
Stage 2	6, 20%	Information on prevention. Topical and oral antibiotics for at least 7–10 days.
Stage 3	2, 6.7%	Information on prevention. Topical and oral antibiotics for 7–10 days and stop wearing the external device for 10–14 days.
Stage 4*	0, 0%	Information on prevention. Parenteral intravenous antibiotics for 7–10 days, surgical intervention, or removal of the infected implanted device.

⁎ The patient with a stage 4 pressure ulcer that required implant removal was referred from another hospital.

**Table 4:** Clinical characteristics of 22 patients with scalp pressure ulcer.

Case No.	Age(years)	Gender	Speech process	Pressure injury stage	POT (month)	medical/skin conditions	Cause of hearing loss
1	3.0	F	Freedom	1	104.59	Healthy	congenital
2	3.25	F	Freedom	1	97.34	Healthy	EVA and Mondini syndrome
3	38.25	F	Freedom	1	75.67	Healthy	progressive
4	34.33	M	Freedom	1	51.57	Healthy	progressive
5	3.18	F	N5	1	0.95	Healthy	EVA
6	1.60	M	N5	3	12.26	ADHD	congenital
				2	50		
7	2.0	F	N5	1	8.89	Healthy	EVA and Mondini syndrome
				1	28.39		
				1	61.44		
8	5.4	M	N5	1	4.79	Ichthyosis	KID syndrome
9	6.81	F	N5	1	18.98	Healthy	EVA
10	2.79	M	N5	2	2.2	ADHD	congenital
				2	62.16		
11	2.91	M	N5	1	2.66	ADHD	congenital
12	2.04	F	N5	2	22.52	Healthy	congenital
13	1.61	M	N6	1	1.44	MDD	congenital
14	1.74	M	N6	2	17.80	ADHD	congenital
15	2.02	M	N6	1	0.89	ADHD	congenital
				1	30.79		
16	60.9	M	N6	1	30.62	Healthy	progressive
17	2.08	M	N6	1	8.13	MR	congenital
				1	28.16		
18	2.42	F	N6	1	0.89	Healthy	congenital
19	3.68	F	N6	1	1.11	Healthy	Pendred syndrome
20	6.97	M	N6	2	19.77	Healthy	congenital
21	28.01	F	N6	1	4.92	Healthy	Waardenburg syndrome
22	45.16	F	N6	1	1.34	MD Schizophrenia	Uremia
				1	11.21		
				3	16.26		

Abbreviations: POT = postoperative time; ADHD = attention deficit hyperactivity disorder; MDD = mixed development disorder; MR = mental retardation; MD = major depression; EVA= enlarged vestibular aqueduct.

## 2 Experimental Design, Materials and Methods

### 2.1 Experimental design

Cochlear implantation (CI) is a proposed safe and effective method for treating congenital or acquired severe and profound sensorineural hearing loss, with a low rate of postoperative complications [3]. The dataset was collected at a single tertiary medical institution from 2001 to 2019. All the patients were diagnosed with bilateral sensorineural severe or profound hearing loss through considerate objective and/or subjective hearing assessments and in the absence of the benefits from amplifications. Those patients were suggested and received the cochlear implants, then were retrospectively included in this research. We excluded the devices other than Nucleus. Besides, due to retrospectively, the patients with mild symptoms and signs may not return to the clinic, thus unable to be included. Using these retrospective data, we had investigated the skin flap infection incidence and further identified the associated causes, and proposed the corresponding managements.

### 2.2 Participants and Setting

We retrospectively reviewed the medical records of 315 adult and pediatric patients who underwent CI in the department of Otolaryngology, Chang Gung Memorial Hospital, between 2001 and 2019. This research was approved by our institutional review board. The need for permission to review medical records retrospectively was waived. The surgery was performed by a single surgeon (C-F, Hwang). Initially, an inverted J skin incision was applied and then accompanied with surgical and instruments progresses, a minimal invasive incision [4]was applied since July 2005. All patients were utilizing intraoperative facial nerve monitor.

### 2.3 Patient and Skin Reaction Evaluation

During routine follow-up visits, some patients with skin reactions over the antenna site were noted. We further determined the skin reaction was similar to pressure injury and related to different antenna designs (Table 2 and fig. 1). The incidence of pressure ulcers was 7.0 % (22/315) in our research. The age of the skin reaction group is younger than the group without skin reaction, despite not significant. Moreover, we used a cutoff of 7 years of age to assess the groups, the incidence was significantly higher in patients ≤ 7 years old than in those > 7 years old.

#### 2.3.1 Skin pressure injury grading and management

There are twenty-two patients, among them six patients had recurrent episodes, that amount to 30 skin pressure injuries after compression by the antenna following CI (Table 4). The complications occurred an average of 26 months (range: 0.89−104.59 months) after surgery. There were five adult and seventeen pediatric patients and their causes of hearing loss and related medical conditions were also detailed in Table 4. Most of the skin reactions were located at the central part of the antenna site and only one (case 15) was located at the peripheral part (fig. 3B). We further identify the risk factor for pediatric cochlear implantation was in the condition of developmental delay with limited language expression, while for adult cochlear implantation was that with irradiated history resulting in compromised circulation over skin flap.

The grade of severity with 1 to 4 scale pressure injury was employed with NPUAP pressure injury staging system (fig. 2) [2], and the corresponding management was proposed (Table 3). The incidence of stage 1 to 4 pressure injury was 73.3 % (22/30), 20.0 % (6/30), 6.7 % (2/30), and 0% (0/0), respectively (Table 3). The management of stage 1 (erythema, slight irritation) pressure injury was suggested to stop wearing the device for a brief period, loosened the magnet or use a coil spacer (fig. 3A) to relieve pressure on the coil, and received topical antibiotics. Stage 2 (skin breakdown, redness and swelling) and stage 3 (full-thickness skin loss) pressure injury were treated with oral antibiotics and the patients with stage 3 injuries were instructed not to wear the external device for 10−14 days. The skin reactions were all subsided with appropriate managements. In our dataset, the stage 4 (exposed bone, muscle, or implant, and infection leading to the removal of the implant) injury (fig. 2D) patient referred by another medical institute was an irradiated nasopharyngeal cancer patient with skin pressure ulcer 19 months after cochlear implantation. His pressure ulcer was visualized with antenna track due to compression by wearing a tight helmet daily. And the patient was successfully treated by transposition of the device and skin flap reconstruction.

#### 2.3.2 Different antenna design

The incidences of skin reactions with different antenna design were 0% with ESPrit speech processor, 18.2 % with Freedom processor, 36.4 % with N5 processor, 45.5 % with N6 processor (Table 2). Moreover, we further noted the protrusion component was associated with skin pressure ulcers. The protrusion of the ESPrit external magnet (0.60 mm) was less pronounced than that of the Freedom (1.28 mm), N5 (1.60 mm), and N6 (0.80 mm) devices and the incidence of this speech processor was significantly lowest [1].

## Ethics Statement

The retrospective research was approved by the institutional review board.

## Declaration of Competing Interest

The authors declare that they have no known competing financial interests or personal relationships which have, or could be perceived to have, influenced the work reported in this article.

## References

[1] Hsieh Hui-Shan, L C.-Y., Wu Hung-Pin, Zhuo Ming-Ying, Hwang Chung-Feng (2020). Pressure ulcers and skin infections after cochlear implantation: a delayed yet serious issue. Int. J. Pediatr. Otorhinolaryngol..

[2] Defloor T., Schoonhoven L., Fletcher J., Furtado K., Heyman H., Lubbers M., Witherow A., Bale S., Bellingeri A., Cherry G. (2005). Statement of the European Pressure Ulcer Advisory Panel-pressure ulcer classification: differentiation between pressure ulcers and moisture lesions. J. Wound Ostomy Continence Nurs..

[3] Terry B., Kelt R.E., Jeyakumar A. (2015). Delayed complications after cochlear implantation. JAMA Otolaryngol. Head Neck. Surg..

[4] Lui C.C., Peng J.P., Li J.H., Yang C.H., Chen C.K., Hwang C.F. (2013). Detection of receiver location and migration after cochlear implantation using 3D rendering of computed tomography. Otol. Neurotol..

